# Molecular and immunological associations of elevated serum lactate dehydrogenase in metastatic melanoma patients: A fresh look at an old biomarker

**DOI:** 10.1002/cam4.3474

**Published:** 2020-10-05

**Authors:** Grant M. Fischer, Fernando C. L. Carapeto, Aron Y. Joon, Lauren E. Haydu, Huiqin Chen, Fuchenchu Wang, John S. Van Arnam, Jennifer L. McQuade, Khalida Wani, John M. Kirkwood, John F. Thompson, Michael T. Tetzlaff, Alexander J. Lazar, Hussein A. Tawbi, Jeffrey E. Gershenwald, Richard A. Scolyer, Georgina V. Long, Michael A. Davies

**Affiliations:** ^1^ Departments of Translational Molecular Pathology The University of Texas MD Anderson Cancer Center Houston TX USA; ^2^ Melanoma Medical Oncology The University of Texas MD Anderson Cancer Center Houston TX USA; ^3^ Biostatistics The University of Texas MD Anderson Cancer Center Houston TX USA; ^4^ Surgical Oncology The University of Texas MD Anderson Cancer Center Houston TX USA; ^5^ University of Pittsburgh Medical Center Pittsburgh PA USA; ^6^ Melanoma Institute of Australia The University of Sydney North Sydney NSW Australia; ^7^ The University of Sydney Sydney NSW Australia; ^8^ Royal Prince Alfred Hospital NSW Health Pathology Sydney NSW Australia; ^9^ Pathology/Lab Medicine The University of Texas MD Anderson Cancer Center Houston TX USA; ^10^ Genomic Medicine The University of Texas MD Anderson Cancer Center Houston TX USA; ^11^ Royal North Shore Hospital Sydney NSW Australia; ^12^ Systems Biology The University of Texas MD Anderson Cancer Center Houston TX USA

**Keywords:** melanoma, molecular profiling, serum lactate dehydrogenase, tumor immunity, tumor metabolism

## Abstract

Elevated serum lactate dehydrogenase (sLDH) is associated with poor clinical outcomes in patients with stage IV metastatic melanoma (MM). It is currently unknown if sLDH elevation correlates with distinct molecular, metabolic, or immune features of melanoma metastases. The identification of such features may identify rational therapeutic strategies for patients with elevated sLDH. Thus, we obtained sLDH levels for melanoma patients with metastases who had undergone molecular and/or immune profiling. Our analysis of multi‐omics data from independent cohorts of melanoma metastases showed that elevated sLDH was not significantly associated with differences in immune cell infiltrate, point mutations, DNA copy number variations, promoter methylation, RNA expression, or protein expression in melanoma metastases. The only significant association observed for elevated sLDH was with the number of metastatic sites of disease. Our data support that sLDH correlates with disease burden, but not specific molecular or immunological phenotypes, in metastatic melanoma.

## Graphical abstract

The association between elevated serum lactate dehydrogenase (sLDH) and poor prognosis in metastatic melanoma patients is well‐established but poorly understood. Our multi‐omics analysis of multiple cohorts of patients with melanoma metastases identified no significant intratumoral molecular, immunological, or metabolic associations with sLDH, supporting that sLDH serves at least partially as a surrogate of tumor burden but not of adverse tumor features.

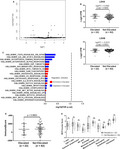

## 1 INTRODUCTION

Lactate dehydrogenase (LDH) is an intracellular enzyme which catalyzes the conversion of pyruvate to lactate and enters the bloodstream following cell death.^1^ In humans, LDH occurs as tetramers consisting of M (or A) subunits encoded by *LDHA* and H (or B) subunits encoded by *LDHB* that assemble to form LDH‐1 (4H), LDH‐2 (3H1 M), LDH‐3 (2H2 M), LDH‐4 (1H3 M), and LDH‐5 (4 M) isoenzymes.^2^ The M subunit preferentially converts pyruvate to lactate while the H subunit catalyzes the reverse reaction. Isoenzymes containing predominantly H subunits tend to predominate in tissues with aerobic metabolism (e.g. heart) while those containing mainly M subunits are found in tissues with considerable anaerobic metabolism (e.g. skeletal muscle and liver).^2^

Initially used to diagnose heart attacks and strokes, serum lactate dehydrogenase (sLDH) became a prominent biomarker in stage IV metastatic melanoma (MM) patients after a series of studies demonstrated a significant association with worse outcomes.^3^, ^4^, ^5^ Both the 7th and 8th editions of the American Joint Committee on Cancer (AJCC) staging system for melanoma incorporate sLDH into the M category criteria for patients with distant metastatic (stage IV) disease.^6^, ^7^, ^8^ Clinical laboratories utilize a colorimetric assay to quickly and sensitively measure total sLDH. While rarely utilized, gel electrophoresis can assess levels of specific isoenzymes.

The association between elevated sLDH and inferior clinical outcomes has persisted despite advances in treatment options.^9^, ^10^, ^11^, ^12^, ^13^, ^14^ Thus, there remains a critical unmet need to develop more effective treatments for patients with elevated sLDH. The development of such treatments may be facilitated by an improved understanding of the pathogenesis of metastatic melanoma with elevated sLDH. We hypothesized that elevated sLDH is associated with molecular and/or immunological features that promote disease progression and/or therapeutic resistance in the tumors of such patients. To test this hypothesis, we performed multi‐omics analyses on cohorts of MM patients with known sLDH values.

## 2 MATERIALS AND METHODS

### 2.1 Patients and specimens

**Figure 1:**
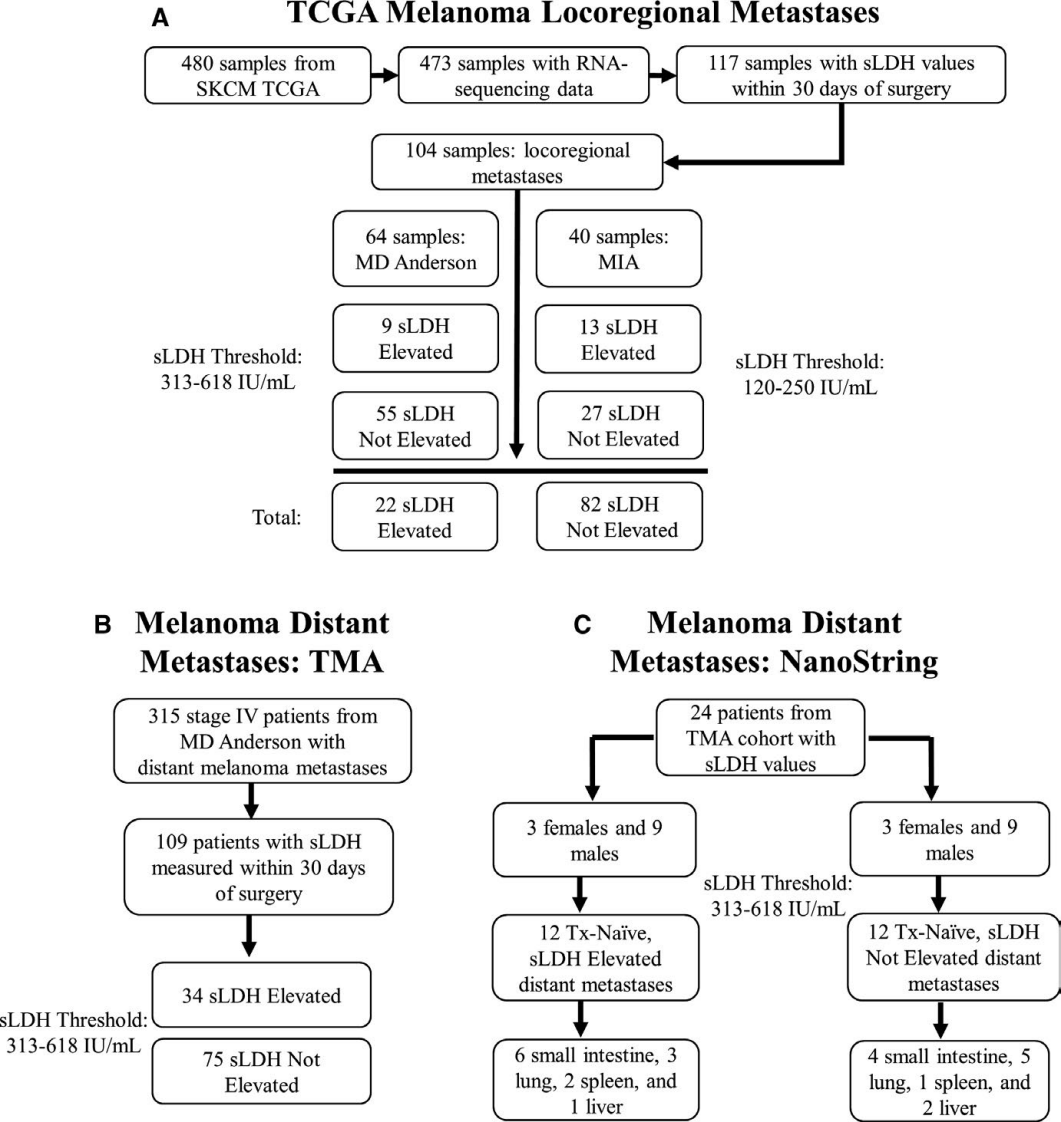
Summary of samples available for molecular profiling. (A) Flowchart diagram illustrating how samples were selected from the Skin Cutaneous Melanoma (SKCM) TCGA. (B‐C) Flowchart diagrams illustrating how distant metastasis samples were selected for analysis via tissue microarray (TMA) and NanoString. MD Anderson, The University of Texas MD Anderson Cancer Center; MIA, Melanoma Institute of Australia.

**Figure 2:**
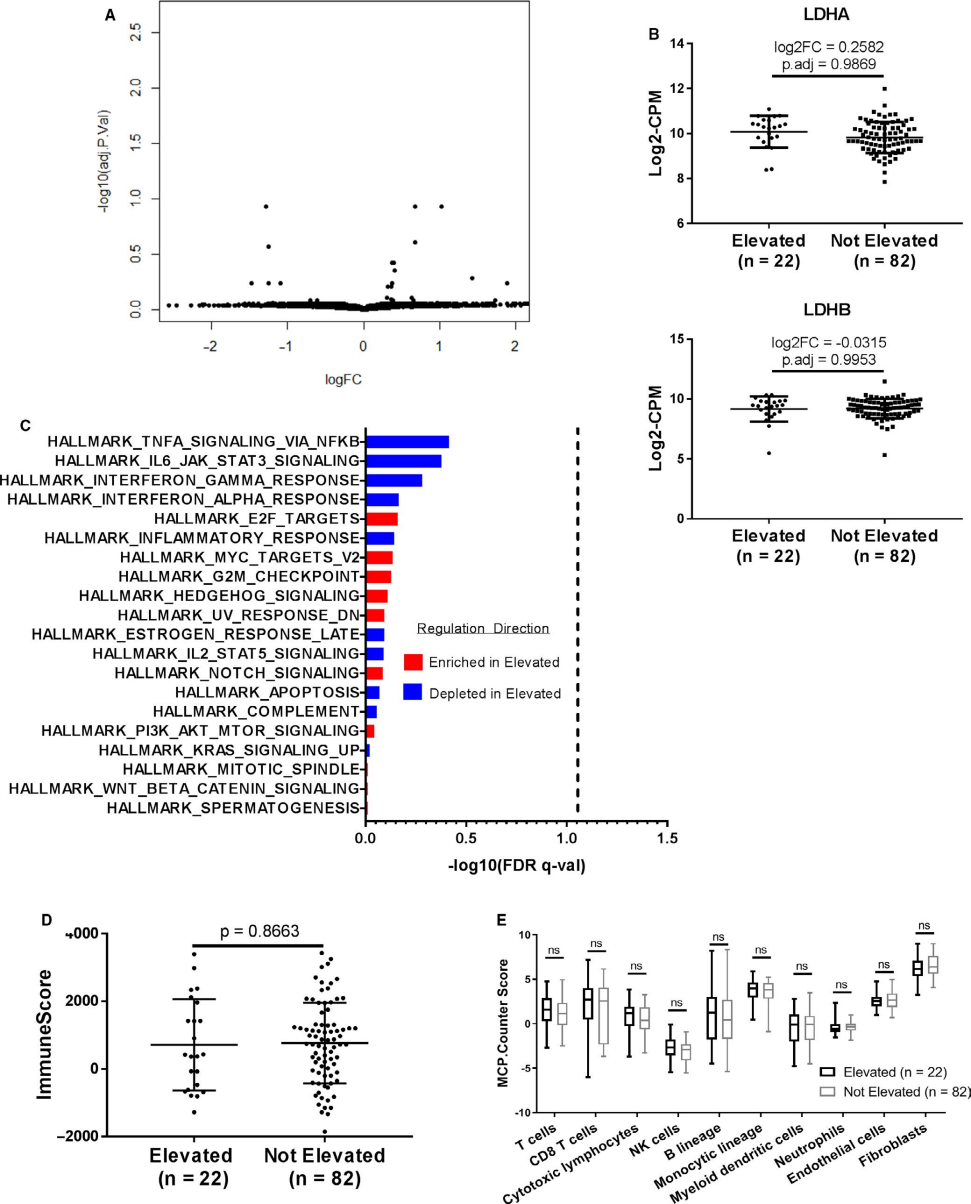
Serum LDH does not associate with molecular or immune features in locoregional melanoma metastases. (A) Volcano plot of limma‐modeled gene expression data between sLDH Elevated (*n* = 22) and sLDH Not Elevated (*n* = 82) TCGA locoregional metastases. The data for all genes are plotted as log2FC vs. the −log10 of the adjusted *p* value. No gene met the criteria for statistical significance (│log2FC│ >1 and adj. *p* value <0.05). (B) Comparison of *LDHA* and *LDHB* gene expression between sLDH Elevated and sLDH Not Elevated TCGA locoregional metastases. Voom‐transformed log2‐counts per million (CPM) values of the *LDHA* and *LDHB* genes are plotted on the y‐axis. Lines represent mean ±S.D. Each dot represents a single sample. (C) Bar plot of the ‐log10(FDR q‐value) of the top 20 MSigDB Hallmarks gene sets from the comparative GSEA analysis of sLDH Elevated and sLDH Not Elevated TCGA locoregional metastases. Gene sets are ordered in descending order of their ‐log10(FDR q‐value). No gene set met the criteria for statistical significance, shown on the graph as a dashed vertical line (FDR q‐value <0.05). (D) ESTIMATE ImmuneScore analysis of sLDH Elevated vs. sLDH Not Elevated TCGA locoregional metastases. Lines represent mean ±S.D., and each dot represents a single sample. Significance determined via two‐sided Student's *t* test. (E) MCP‐Counter analysis of sLDH Elevated vs. sLDH Not Elevated TCGA locoregional metastases. Each plot is a simple box and whisker plot. Median values (lines) and interquartile range (whiskers) are indicated. ns: not significant (*p* > 0.05) by two‐sided Student's *t* test.

Two cohorts of melanoma metastases from patients with sLDH levels measured within 30 days of surgery were included in the study: (1) locoregional (skin and regional lymph node) metastases from patients included in The Cancer Genome Atlas's (TCGA’s) skin cutaneous melanoma (SKCM) database with RNA‐sequencing (RNA‐seq) (*n* = 104), whole exome sequencing (WES) (*n* = 92), DNA methylation (*n* = 104), and reverse phase protein array (RPPA) (*n* = 71) data (Figure 1), and (2) distant metastases from stage IV MM patients treated at The University of Texas MD Anderson Cancer Center (MD Anderson) between 1998 and 2010. For the latter cohort, tumors were obtained from the MD Anderson Melanoma Informatics, Tissue Resource, and Translational Pathology Core (MelCore) under an Institutional Review Board‐approved protocol. Formalin fixed, paraffin‐embedded (FFPE) tissue was used to construct a tissue microarray (TMA) for immunohistochemistry (IHC) studies (*n* = 109) and to isolate RNA for measurement of metabolic and immunological genes (*n* = 24) by NanoString nCounter (Figure 1).

### 2.2 sLDH classification

sLDH values were collected from clinical records for patients included in the SKCM TCGA from MD Anderson and the Melanoma Institute of Australia (MIA). The use of a local reference (upper limit of normal, or ULN) is the convention used to categorize sLDH values in the melanoma AJCC staging system. Thus, sLDH values from MD Anderson patients were considered “Elevated” if values were greater than 618 IU/mL, the ULN (313‐618 IU/mL) for the MD Anderson clinical chemistry laboratory. sLDH values from MIA patients were considered “Elevated” if values were greater than 250 IU/mL, the ULN (120‐250 IU/mL) for the MIA clinical chemistry laboratory (Figure 1).

### 2.3 TCGA analyses

For all locoregional metastases in the SKCM TCGA with available sLDH values, publicly available molecular data were downloaded from the GDAC Firehose, including: raw expected counts generated via RSEM (sLDH Elevated, *n* = 22; sLDH Not Elevated, *n* = 82) and level 3 somatic mutations (sLDH Elevated, *n* = 18; sLDH Not Elevated, *n* = 74); CNV (sLDH Elevated, *n* = 18; sLDH Not Elevated, *n* = 74); DNA methylation (sLDH Elevated, *n* = 22; sLDH Not Elevated, *n* = 82); and RPPA (sLDH Elevated, *n* = 16; sLDH Not Elevated, *n* = 55). Details of analyses are provided in Data S1.

### 2.4 TMA

One hundred and nine distant melanoma metastases were included in the construction of the TMA (Figure 1 and Table S1). Each sample was represented by two 1 mm cores prepared on a total of five FFPE blocks.

TMA samples underwent immunohistochemistry (IHC) staining for M (or A) subunits of LDH, H (or B) subunits of LDH, PTEN, PD‐L1, MITF, CD8, and Ki67. LDHA subunit (ABCAM cat. #AB 84716, 1:300), LDHB subunit (ABCAM cat. #AB 85319, 1:25,000), PTEN (6H2.1 clone, DAKO, 1:100), Ki67 (Cell Signaling cat. #9027, 1:400), and CD8 (Life Technology cat. #MS457, 1:25) were stained in the Leica bond RX IHC stainer. PD‐L1 (Dako clone 28‐8 and clone 22C3) and MITF (ThermoFisher Scientific clone D5) were stained in the MD Anderson pathology department within a clinical laboratory improvement amendments (CLIA) environment.^15^ All slides were digitalized with the Leica scanner. Tumor cell LDHA and LDHB were scored as 0 (negative), 1 (mild/moderate), or 2 (intense). Tumor cell PTEN was scored as present or absent, as previously described.^16^ PD‐L1, Ki67, and MITF were scored as the percentage of tumor cells with positive membranous (PD‐L1) or nuclear (Ki67 and MITF) staining per 1 mm^2^. Positivity for CD8 was scored as a percentage of intratumoral lymphocytes showing membranous positivity per 1 mm^2^. All IHC was scored by two pathologists (FCLC and JSV). Examples of staining patterns are provided in Figure S1.

### 2.5 NanoString gene expression analysis

A total of 24 treatment‐naive samples (sLDH Elevated, n = 12; sLDH Not Elevated, n = 12) balanced for gender and accession site were selected from the TMA samples to undergo additional molecular profiling (Figure 1). RNA was isolated as previously described.^17^ Gene expression data were collected for analysis via the NanoString nCounter Vantage 3D Cancer Metabolism Panel and NanoString nCounter PanCancer Immune Profiling Panel and analyzed via nSolver™ Analysis Software. Details of analyses are provided in Data S1.

## 3 RESULTS

### 3.1 Lack of significant association of molecular and immune features with sLDH status in locoregional metastases from the cutaneous melanoma TCGA

RNA‐seq data were collected for regional metastases from the SKCM TCGA with sLDH values measured within 30 days of surgical accession (*n* = 104) (Figure 1). Age, sex, BRAF mutation status, and prior treatment status did not differ significantly between the sLDH Elevated (*n* = 22) and sLDH Not Elevated patients (*n* = 82) (Table S2). Histologically, tumors from patients with elevated sLDH and those from patients without elevated sLDH did not differ significantly in extent of pigment (*p* = 0.1753), cell size (*p* = 0.5497), cell shape (*p* = 0.5823) (Table S3), tumor cell content (*p* = 0.3700), or necrosis (*p* = 0.7382) (Figure S2).

We performed principal component analysis (PCA) of the 1,000 most variable genes to elucidate transcriptomic heterogeneity among the groups. No distinct clusters formed between sLDH Elevated and sLDH Not Elevated samples, indicating overall similarity between groups (Figure S3). Comparative RNA‐seq analysis identified 159 genes with nominal *p* values <0.05 and │log2FCs│ >1 between the groups; however, no genes were statistically significant after correcting for multiple hypothesis testing (Figure 2A). Notably, neither *LDHA* (*p*.adj=0.9869) nor *LDHB* (*p*.adj=0.9953) expression correlated with sLDH status (Figure 2B), indicating that tumor cell expression of these genes did not explain differences in sLDH levels. Additionally, pathway analysis failed to identify significant differences in the MSigDB Hallmarks gene set collection between sLDH Elevated and sLDH Not Elevated tumors (Figure 2C). Given the positive associations observed between immune infiltrates and clinical outcomes in the SKCM TCGA and immunotherapy clinical trials,^18^, ^19^, ^20^ we utilized the ESTIMATE^21^ and MCP‐Counter^22^ R packages to assess the immune cell infiltration in the tumors. Comparative analysis identified no significant differences in immune cell infiltration either via ESTIMATE analysis (*p* = 0.8663) (Figure 2D) or MCP‐Counter analysis (*p* > 0.05 for all immune cell populations) between tumors from patients with elevated sLDH and those from patients without elevated sLDH (Figure 2E).

Comparative analysis of WES data identified no significant differences in copy number variation profiles, somatic mutation burden, or somatic mutation rate across 72 therapeutically targetable genes between tumors from patients with sLDH Elevated (*n* = 18) vs. tumors from patients without elevated sLDH (*n* = 74) (Figure S4A‐C). Global DNA methylation analysis identified only three probes with nominal *p* values <0.05; none of these probes remained significant following correction for multiple hypothesis testing (Figure S5). Finally, analysis of proteomic data from RPPA identified only three proteins with significantly different expression (nominal *p* values <0.05) between sLDH Elevated (*n* = 16) and sLDH Not Elevated (*n* = 54) tumors, which were no longer significant following correction for multiple hypothesis testing (Table S4). RPPA pathway analysis also failed to identify significant differences in any of the 12 pathways (Table S4).

### 3.2 Lack of molecular or immunological associations with sLDH status in distant metastases

A total of 109 distant melanoma metastases with sLDH levels assessed within 30 days of tumor resection were evaluated (Figure 1). As predicted, univariate analysis demonstrated that patients with elevated sLDH (*n* = 34) had a significantly greater number of sites of metastatic involvement compared to patients without elevated sLDH (*n* = 75), using multiple thresholds (≥ 3, *p* < 0.0001; ≥4, *p* = 0.0011; ≥5, *p* = 0.0038) (Table 1). Age, sex, BRAF mutation status, and prior treatment status did not differ significantly between the groups (Table S5).

**Table 1:** Total Number of Metastatic Sites in TMA Patients

# Metastatic Sites	Serum LDH (n = 109)		Fisher test *p* value
	Elevated (n = 34)	Not Elevated (n = 75)	
≥ 3	16	7	<0.0001
≥ 4	10	4	0.0011
≥ 5	7	2	0.0038

The cumulative number of metastatic sites confirmed in TMA patients within 30 days of sLDH assessment were compared between patients with elevated sLDH (*n* = 34) and patients without elevated sLDH (*n* = 75). Significantly more patients with elevated sLDH had a high metastatic burden, regardless of the threshold used to define “high metastatic burden” (at least 3, 4, or 5 sites of metastasis).

**Figure 3:**
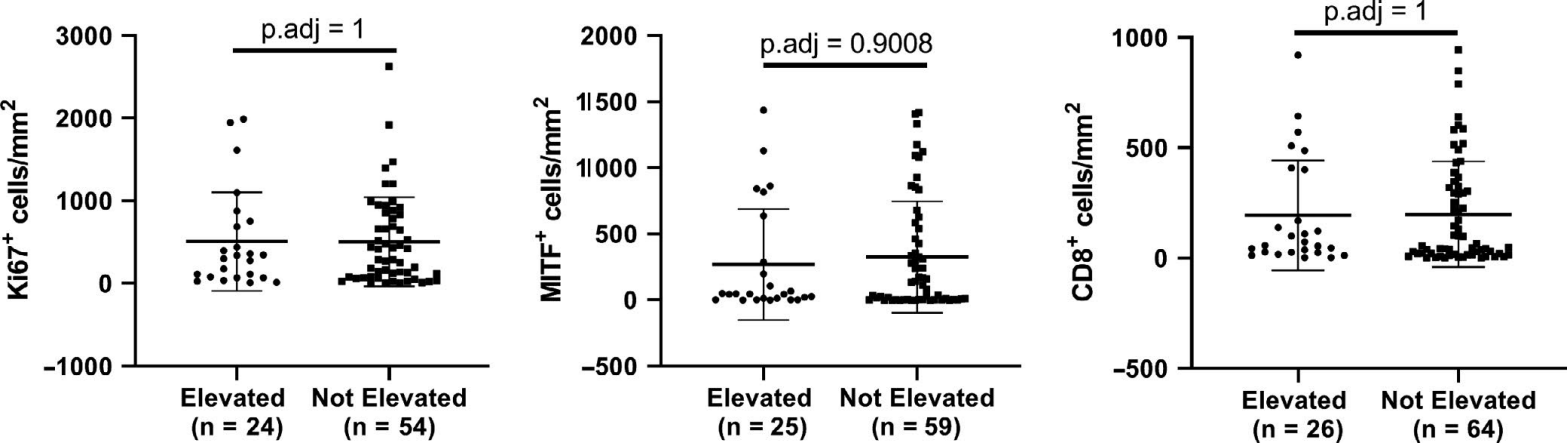
Serum LDH does not associate with TMA markers evaluated as continuous variables in distant melanoma metastases. Ki67, MITF, and CD8 positivity do not differ between distant metastases acquired from patients with elevated sLDH and distant metastases taken from patients without elevated sLDH. Lines represent mean ±S.D., and each dot represents a single sample. Significance determined via two‐sided Student's *t* test.

Consistent with our analysis of TCGA RNA‐seq data, we observed no significant differences in the protein expression (by IHC) of LDHA (*p*.adj=0.8837) or LDHB (*p*.adj=1) subunits between tumors from melanoma patients with sLDH Elevated vs. Not Elevated (Table 2). We analyzed tumor proliferation, with the rationale that more proliferative tumors would have a greater amount of cellular turnover, leading to an increased release of LDH into the bloodstream. However, Ki67 staining did not differ between the groups (*p*.adj=1) (Figure 3). MITF, a lineage‐specific pro‐survival gene in melanoma, also did not differ between groups (*p*.adj=0.9008) (Figure 3). Additionally, we evaluated loss of PTEN, given its prognostic significance in MM and role in mediating immunosuppression.^16^, ^23^ Comparative analysis initially determined that complete loss of PTEN occurred significantly more often in sLDH Elevated tumors (*p* = 0.0415), but we noted no significant difference in the prevalence of complete PTEN loss between groups following adjustment for multiple hypothesis testing (*p*.adj=0.3320) (Table 2). Finally, neither PD‐L1 positivity (*p*.adj=1 and *p*.adj=0.7228 for clones 28‐8 and 22C3, respectively) (Table 2) nor CD8^+^ immune infiltrates (*p*.adj=1) (Figure 3) associated significantly with sLDH status.

**Table 2:** Melanoma TMA Results of Categorically Scored Markers

Marker	Serum LDH			*p* value		Adj. *p* value
	Elevated n (%)		Not Elevated n (%)			
LDHA						
0		3 (9)	13 (17)			
1		23 (68)	51 (68)		0.3314	0.8837
2		8 (24)	11 (15)			
Total		34	75			
LDHB						
0		0 (0)	2 (3)			
1		5 (20)	9 (16)		0.9078	1
2		20 (80)	47 (81)			
Total		25	58			
PTEN						
Absent		11 (32)	11 (15)		0.0415	0.3320
Total		34	75			
PD‐L1 clone 28‐8						
Positive (>5%)		8 (24)	10 (13)		0.2643	1
Total		34	75			
PD‐L1 clone 22C3						
Positive (>1%)		11 (32)	18 (24)		0.3614	0.7228
Total		34	75			

The TMA analysis did not identify significant intratumoral alterations (adj. *p* value <0.05) in any of the listed markers between sLDH Elevated and sLDH Not Elevated distant metastases. Each marker is listed along with the scoring mechanism used for the analysis. Scores for samples are listed along with the total number of samples analyzed by each marker. Only markers scored categorically are included in the table.

### 3.3 Lack of association between metabolic and immunological gene expression and sLDH status in distant melanoma metastases

**Figure 4:**
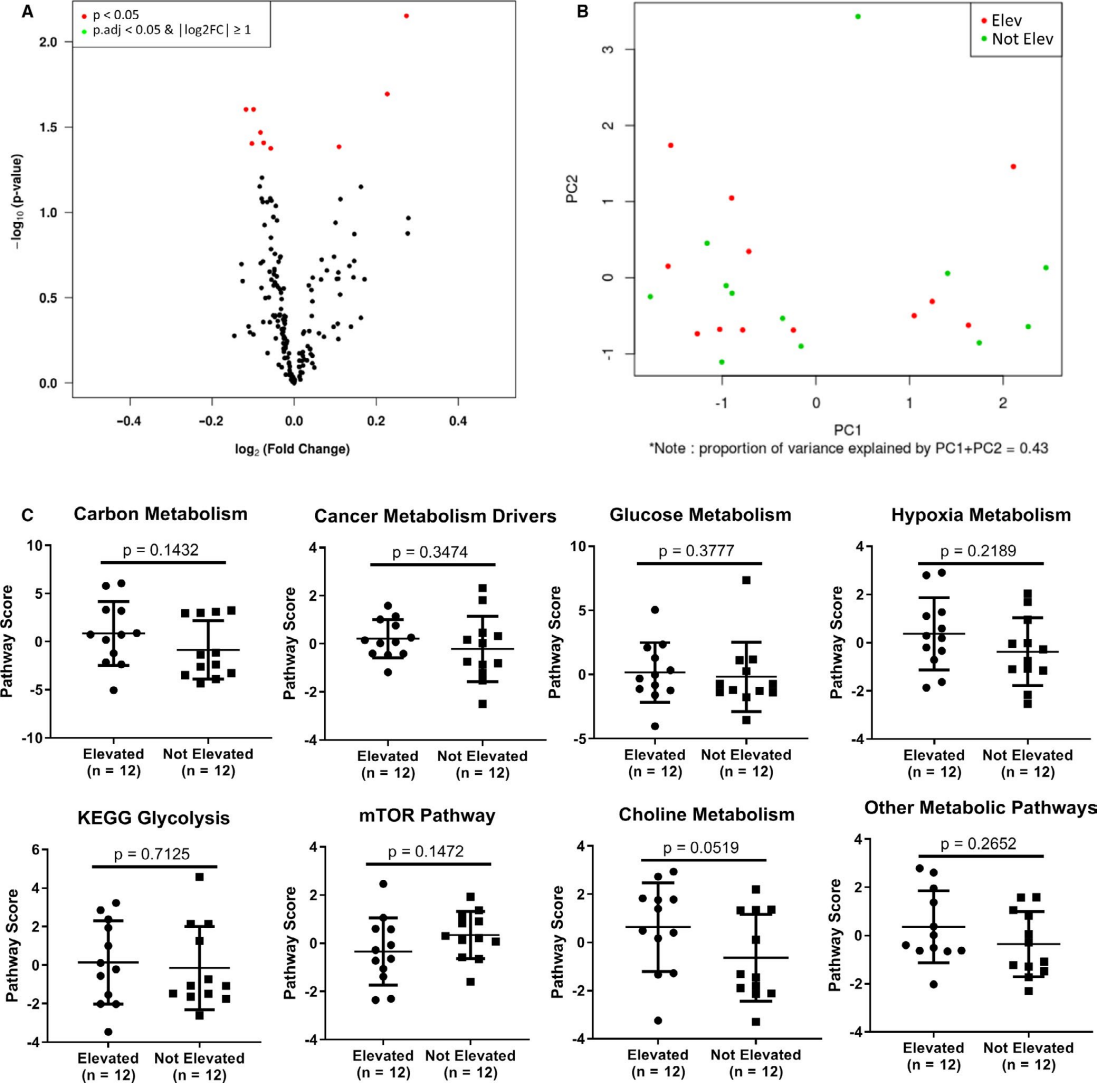
Serum LDH does not associate with metabolic features in distant melanoma metastases. (A) Volcano plot of normalized NanoString nCounter Vantage 3D Cancer Metabolism Panel gene expression data for 12 distant melanoma metastases from patients with elevated sLDH and 12 distant melanoma metastases from patients without elevated sLDH. The data for all genes are plotted as log2FC vs. the −log10 of the adjusted *p* value. No gene met the criteria for statistical significance (│log2FC│ >1 and adj. *p* value <0.05). (B) PCA plot analysis of normalized NanoString nCounter Vantage 3D Cancer Metabolism Panel gene expression data. No obvious grouping was observed. (C) Pathway analysis of NanoString nCounter Vantage 3D Cancer Metabolism Panel gene expression data. Increasing pathway scores correspond to increasing expression. Lines represent mean ±S.D., and each dot represents a single sample. Significance determined via two‐sided Student's *t* test. No pathway met the criteria for statistical significance (*p* < 0.05).

Recent work has demonstrated that metabolic pathways in tumors can play critical roles in resistance to targeted and immune therapies in MM patients.^24^, ^25^, ^26^ Thus, we selected 24 treatment‐naïve distant metastases (*n* = 12 sLDH Elevated and *n* = 12 sLDH Not Elevated) from the 109 TMA samples for focused gene expression analysis using the NanoString nCounter Vantage 3D Cancer Metabolism Panel. Samples were selected to ensure an equal balance of gender and site of distant metastasis between groups (Figure 1). A total of 180 genes from various metabolic pathways were assessed. No genes met the criteria for statistical significance following correction for multiple hypothesis testing (Figure 4A). Further, sLDH Elevated and sLDH Not Elevated samples did not form distinct clusters following PCA analysis of the normalized expression data (Figure 4B). Finally, we observed no significant differences between groups for the following pathways: carbon metabolism, cancer metabolism drivers, glucose metabolism, hypoxia metabolism, KEGG glycolysis, mTOR pathway, choline metabolism, or other metabolic pathways (Figure 4C).

**Figure 5:**
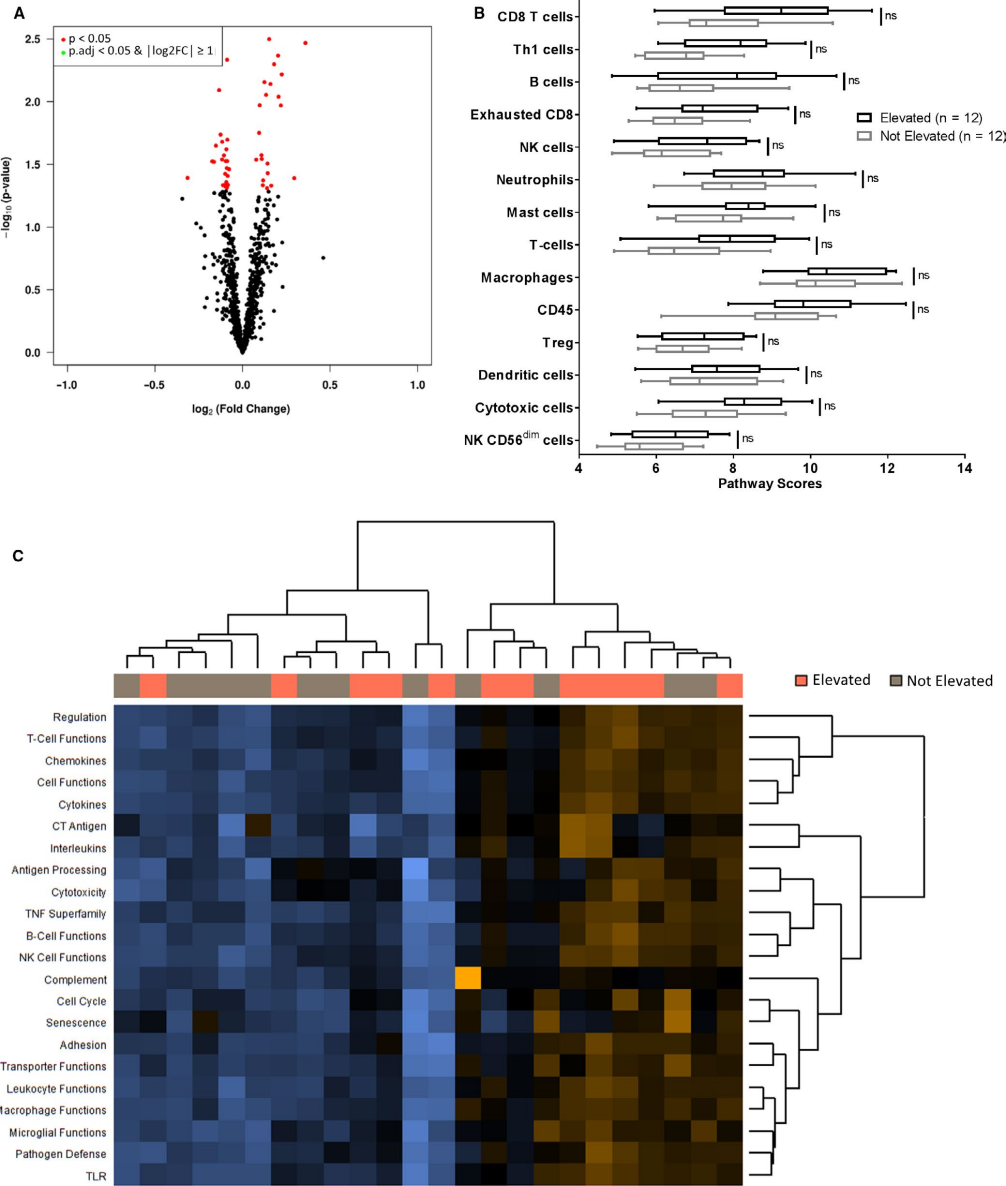
Serum LDH does not associate with immune features in distant melanoma metastases. (A) Volcano plot of normalized NanoString nCounter PanCancer Immune Profiling Panel gene expression data from 12 distant melanoma metastases from patients with elevated sLDH and 12 distant melanoma metastases from patients without elevated sLDH. The data for all genes are plotted as log2FC vs. the −log10 of the adjusted *p* value. No gene met the criteria for statistical significance (│log2FC│ >1 and adj. *p* value <0.05). (B) Intratumoral immune cell analysis of sLDH Elevated (*n* = 12) and sLDH Not Elevated (*n* = 12) distant melanoma metastases. Each plot is a simple box and whisker plot. Median values (lines) and interquartile range (whiskers) are indicated. ns: not significant (*p*.adj>0.05) by two‐sided Student's *t* test. (C) Pathway analysis of NanoString nCounter PanCancer Immune Profiling Panel gene expression data. Hierarchical clustering analysis failed to identify obvious grouping between sLDH Elevated (*n* = 12) and sLDH Not Elevated samples (*n* = 12).

RNA from the tumors was also assessed using the NanoString nCounter PanCancer Immune Profiling Panel, which includes 730 genes reflecting immune cell types, common checkpoint inhibitors, cancer/testis antigens, and genes covering both the adaptive and innate immune response. No statistically significant differences in expression between metastatic melanomas from patients with sLDH Elevated vs. Not Elevated were noted (Figure 5A). We observed no differences in intratumoral estimates of any immune cell subpopulation (Figure 5B) or any immunological pathway available for analysis (Figure S6). Further, hierarchical clustering of samples by these pathways did not yield distinct clusters between sLDH Elevated and sLDH Not Elevated samples (Figure 5C).

## 4 DISCUSSION

The association between elevated sLDH and worse outcomes in MM patients has been observed for over two decades.^3^, ^4^, ^5^ Several recent studies have determined that elevated sLDH associates with poor outcomes in clinical trials of targeted and immune therapies. For example, Schadendorf et al. demonstrated that elevated baseline sLDH independently associated with decreased progression free survival (PFS) and overall survival (OS) in MM patients treated with dabrafenib and trametinib.^27^ Likewise, studies by both Wagner et al. and Joseph et al. reported that elevated sLDH at baseline associated with worse OS in MM patients treated with anti‐PD1.^28^, ^29^ Here we have utilized cohorts of molecularly characterized and clinically annotated melanoma metastases to study the mechanisms that may underlie these associations.

To our knowledge, this is the first study to comprehensively assess the associations of molecular or immune features of melanoma metastases with elevated sLDH. Our analysis of multiple cohorts, including multi‐omic data from the cutaneous melanoma TCGA, showed that there were minimal differences in molecular, immune, and metabolic features between metastases from patients with elevated sLDH vs. not elevated sLDH. Thus, our data support that the poor clinical outcomes associated with sLDH levels cannot be readily attributed to differences in specific immune, molecular, or metabolic features.

Our transcriptomic analysis of locoregional metastases from the TCGA database demonstrated global similarity between metastases from sLDH Elevated and sLDH Not Elevated melanoma patients. NanoString analyses of distant metastases corroborated that sLDH did not associate with differences in metabolic pathways. In contrast to a study published by Huang et al.^30^, our analyses by bulk RNA‐seq and Nanostring did not identify differences in immune function between groups. However, additional (i.e. single‐cell) analyses could be done to interrogate this specifically. Likewise, proteomic analysis did not identify significant differences in individual proteins or pathways between locoregional metastases from patients with elevated sLDH and those from patients without elevated sLDH. Our TMA IHC analysis also identified no correlation between LDHA, LDHB, PTEN, MITF, CD8, Ki67, and – similar to Joseph et al.^28^ – PD‐L1 staining and sLDH status in distant metastases. WES analyses of locoregional metastases identified no copy number alterations or differences in mutation burden between sLDH Elevated and sLDH Not Elevated groups. Finally, methylation studies did not identify differentially methylated probes between sLDH Elevated and sLDH Not Elevated locoregional metastases. The cohorts included a relatively large number of samples, and with their deep molecular and immune profiling, they represent one of the largest and most comprehensive datasets to test for intratumoral features associated with sLDH. While the lack of statistical significance observed could be attributed to inadequate sample size, we believe that the consistent lack of difference across all analyses is highly suggestive that any differences, should they exist, would not correlate with the magnitude of difference in clinical outcomes between patients with elevated sLDH vs. not elevated sLDH.

Our findings address several gaps in our current understanding of the pathogenesis of elevated sLDH in melanoma. First, elevated sLDH is not attributable to increased expression of LDHA and LDHB by tumor cells, nor does it reflect increased cellular proliferation or death. Second, the poor prognosis associated with elevated sLDH cannot be attributed to increased expression of genes or pathways known to mediate a more aggressive phenotype or therapeutic resistance in this disease. For example, while enrichment of the glycolytic pathway mediates resistance to adoptive T cell therapy in MM, and oxidative phosphorylation can cause resistance to targeted therapies and anti‐PD1,^25^, ^26^, ^31^ neither of these metabolic pathways were enriched in metastases from patients with elevated sLDH. Similarly, proteomics analysis ruled out oncogenic signaling pathways (i.e. PI3 K‐AKT) as potential mediators of poor prognosis in patients with elevated sLDH.^16^ Finally, genetic and epigenetic features do not likely explain the association between elevated sLDH and poor prognosis / therapeutic resistance.^32^, ^33^

It is possible that sLDH’s association with worse outcomes solely relates to it being a surrogate for tumor burden. Indeed, we found a strong association between elevated sLDH and total number of metastatic sites of disease in this study. Likewise, Joseph et al. quantified baseline tumor size (BTS) using RECIST criteria target lesions and found a significant correlation between sLDH and this measure.^28^ Huang et al. also reported that sLDH correlated with tumor burden in their cohort of MM patients.^30^ Despite these consistent associations, some multivariate analyses have also shown that sLDH can correlate with worse outcomes independent of tumor burden.^3^, ^4^ Notably, Joseph et al. demonstrated that sLDH was independently associated with overall response rate (ORR) to anti‐PD1, but BTS was not (although the actual results for BTS were not available for further interrogation).^28^ Results like these suggest that tumor burden may only partly explain sLDH’s prognostic value.

Notably, there are additional possible contributors to sLDH levels that are independent of tumor burden. As LDH is a widely distributed enzyme, elevated levels in the bloodstream can result from damage to nonmalignant tissues (i.e., heart, liver, kidneys, and red blood cells).^2^ Therefore, baseline sLDH levels may differ between MM patients in part due to differential organ injury and enzyme leakage following metastasis. Tumor burden would certainly mediate damage to normal tissues to some extent. However, small invasive metastases, potentially even including micro‐metastases, could damage tissues as well, and could do so without being accounted for by tumor burden measurements in studies applying RECIST criteria to assess tumor burden.^28^ Common benign diseases affecting the heart, liver, or kidneys could cause confounding increases in sLDH in MM patients.^34^, ^35^ It is possible that a subset of MM patients with elevated sLDH do poorly because they are generally less healthy than their counterparts.

It is also possible that sLDH levels in cancer patients could be affected by coagulopathies, due to malignancy and/or cancer treatments.^36^ Disseminated intravascular coagulation (DIC), hemolytic uremic syndrome (HUS), and thrombotic thrombocytopenic purpura (TTP) are the main types of thrombotic microangiopathies seen in cancer patients.^36^ Importantly, these conditions cause intravascular hemolysis, which results in increased sLDH due to rupture of red blood cells.^36^ To our knowledge, no studies of sLDH have incorporated markers of cardiac, liver, renal, or coagulation function into their statistical models, which could be investigated in the future. Further, spontaneous hemorrhage is a common complication of melanoma brain metastases.^37^ Various mechanisms have been proposed to explain intratumoral hemorrhage, including blood vessel wall compression and necrosis due to the expanding tumor, invasion of the blood vessel wall by the tumor, and fragility of the structurally unsound and immature blood vessels.^37^ Each mechanism features varying degrees of endothelial cell injury, which could also elevate sLDH. While previous studies have incorporated sites of metastasis into their statistical models, to our knowledge no study has accounted for intratumoral hemorrhage in MM patients with brain metastases, which could be addressed in future studies. Moreover, future studies should evaluate whether or not sLDH values associate with distinct tumor or clinical features in melanoma brain metastases as this study focused exclusively on nonbrain melanoma metastases.

In conclusion, this study represents the most comprehensive effort to date to improve our understanding of the pathogenesis that underlies the poor clinical outcomes associated with elevated sLDH in MM patients. Taken together, our data support that sLDH serves at least partially as a surrogate for tumor burden, but not for the molecular, immune, or metabolic status of patients’ tumors. The development of more effective treatment strategies remains a key challenge for patients with distant metastatic melanoma, particularly for those with elevated sLDH.

## CONFLICTS OF INTEREST

MAD has been a consultant to Roche/Genentech, Array, Novartis, BMS, GSK, Sanofi‐Aventis, and Vaccinex, and he has been the PI of funded research grants to his institution by Roche/Genentech, GSK, Sanofi‐Aventis, Merck, Myriad, and Oncothyreon. MTT has served on advisory committees for Novartis, Myriad Genetics, and Seattle Genetics. AJL has served on advisory committees and/or scientific advisory boards for BMS, GSK/Novartis, Roche/Genentech, MedImmune/AstraZeneca, Bayer, Guardant and ArcherDX. JEG has served on advisory committees and/or scientific advisory boards for Merck, Novartis, and Syndax. HAT has been a consultant to Roche/Genentech, Array, Novartis, BMS, Merck, and he has been the PI of funded research grants to his institution by Roche/Genentech, Novartis, BMS, Merck, GSK, and Celgene. RAS has received fees for professional services from Merck Sharp & Dohme, GlaxoSmithKline Australia, Bristol‐Myers Squibb, Dermpedia, Novartis Pharmaceuticals Australia Pty Ltd, Myriad, NeraCare, and Amgen. No potential conflicts of interest were disclosed by the other authors.

## AUTHORS’ CONTRIBUTIONS

**Concept and design:** GMF, FCLC, LEH, JSV, JLM, AJL, MTT, and MAD. **Acquisition of data:** GMF, FCLC, LEH, JSV, JLM, KW, JMK, AJL, JFT, MTT, RAS, GVL, and MAD. **Analysis and interpretation of data:** GMF, FCLC, JSV, AYJ, HC, FW, JLM, and MAD. **Writing, review, and/or revision of the manuscript:** All authors. **Study supervision:** GMF, FCLC, MAD.

## Supporting information

Fig S1

Fig S2

Fig S3

Fig S4

Fig S5

Fig S6

Table S1

Table S2

Table S3

Table S4

Table S5

Supplementary Material

## Data Availability

The data that support the findings of this study are available from the corresponding author upon reasonable request.

## References

[1] Palmer SR , Erickson LA , Ichetovkin I , Knauer DJ , Markovic SN . Circulating serologic and molecular biomarkers in malignant melanoma. Mayo Clin Proc. 2011;86:981–990. 21964175 10.4065/mcp.2011.0287 PMC3184027

[2] Kopperschlager G , Kirchberger J . Methods for the separation of lactate dehydrogenases and clinical significance of the enzyme. J Chromatogr B Biomed Appl. 1996;684:25–49. 8906464 10.1016/0378-4347(96)00133-8

[3] Eton O , Legha SS , Moon TE , et al. Prognostic factors for survival of patients treated systemically for disseminated melanoma. J Clin Oncol. 1998;16:1103–1111. 9508197 10.1200/JCO.1998.16.3.1103

[4] Sirott MN , Bajorin DF , Wong GYC , et al. Prognostic factors in patients with metastatic malignant melanoma. A multivariate analysis Cancer. 1993;72:3091–3098. 8221576 10.1002/1097-0142(19931115)72:10<3091::aid-cncr2820721034>3.0.co;2-v

[5] Deichmann M , Benner A , Bock M , et al. S100‐Beta, melanoma‐inhibiting activity, and lactate dehydrogenase discriminate progressive from nonprogressive American Joint Committee on Cancer stage IV melanoma. J Clin Oncol. 1999;17:1891–1896. 10561230 10.1200/JCO.1999.17.6.1891

[6] Gershenwald JE , Scolyer RA , Hess KR , et al. Melanoma staging: Evidence‐based changes in the American Joint Committee on Cancer eighth edition cancer staging manual. CA Cancer J Clin. 2017;67(6):472–492. 29028110 10.3322/caac.21409 PMC5978683

[7] Balch CM , Gershenwald JE , Soong S‐J , et al. Final version of 2009 AJCC melanoma staging and classification. J Clin Oncol. 2009;27:6199–6206. 19917835 10.1200/JCO.2009.23.4799 PMC2793035

[8] Goldberg E , Hawtrey C . The ontogeny of sperm specific lactate dehydrogenase in mice. J Exp Zool. 1967;164:309–316. 6068185 10.1002/jez.1401640302

[9] Robert C , Grob JJ , Stroyakovskiy D , et al. Five‐year outcomes with dabrafenib plus trametinib in metastatic melanoma. N Engl J Med. 2019;381:626–636. 31166680 10.1056/NEJMoa1904059

[10] Hauschild A , Larkin J , Ribas A , et al. Modeled prognostic subgroups for survival and treatment outcomes in BRAF V600‐mutated metastatic melanoma: pooled analysis of 4 randomized clinical trials. JAMA Oncol. 2018;4:1382–1388. 30073321 10.1001/jamaoncol.2018.2668 PMC6233771

[11] Dummer R , Ascierto PA , Gogas HJ , et al. Overall survival in patients with BRAF‐mutant melanoma receiving encorafenib plus binimetinib versus vemurafenib or encorafenib (COLUMBUS): a multicentre, open‐label, randomised, phase 3 trial. Lancet Oncology. 2018;19:1315–1327. 30219628 10.1016/S1470-2045(18)30497-2

[12] Koguchi Y , Hoen HM , Bambina SA , et al. Serum immunoregulatory proteins as predictors of overall survival of metastatic melanoma patients treated with ipilimumab. Can Res. 2015;75:5084–5092. 10.1158/0008-5472.CAN-15-2303 26627641

[13] Wolchok JD , Chiarion‐Sileni V , Gonzalez R , et al. Overall survival with combined nivolumab and ipilimumab in advanced melanoma. N Eng J Med. 2017;377:1345–1356. 10.1056/NEJMoa1709684 PMC5706778 28889792

[14] Weide B , Martens A , Hassel JC , et al. Baseline biomarkers for outcome of melanoma patients treated with pembrolizumab. Clin Cancer Res. 2016;22:5487–5496. 27185375 10.1158/1078-0432.CCR-16-0127 PMC5572569

[15] Fetsch PA , Abati A . The clinical immunohistochemistry laboratory: regulations and troubleshooting guidelines. Methods Mol Biol. 2010;588:399–412. 20012854 10.1007/978-1-59745-324-0_43

[16] Bucheit AD , Chen G , Siroy A , et al. Complete loss of PTEN protein expression correlates with shorter time to brain metastasis and survival in stage IIIB/C melanoma patients with BRAFV600 mutations. Clin Cancer Res: An Official Journal of the American Association for Cancer Research. 2014;20:5527–5536. 10.1158/1078-0432.CCR-14-1027 PMC4216767 25165098

[17] Kwong LN , De Macedo MP , Haydu L , et al. Biological validation of RNA sequencing data from formalin‐fixed paraffin‐embedded primary melanomas. JCO Precision Oncology. 2018;2:1–19. 31058252 10.1200/PO.17.00259 PMC6498859

[18] Tumeh PC , Harview CL , Yearley JH , et al. PD‐1 blockade induces responses by inhibiting adaptive immune resistance. Nature. 2014;515:568–571. 25428505 10.1038/nature13954 PMC4246418

[19] Chen P‐L , Roh W , Reuben A , et al. Analysis of immune signatures in longitudinal tumor samples yields insight into biomarkers of response and mechanisms of resistance to immune checkpoint blockade. Cancer Discov. 2016;6:827–837. 27301722 10.1158/2159-8290.CD-15-1545 PMC5082984

[20] Cancer Genome Atlas N . Genomic classification of cutaneous melanoma. Cell. 2015;161:1681–1696. 26091043 10.1016/j.cell.2015.05.044 PMC4580370

[21] Yoshihara K , Shahmoradgoli M , Martínez E , et al. Inferring tumour purity and stromal and immune cell admixture from expression data. Nature. Communications. 2013;4: 10.1038/ncomms3612 PMC3826632 24113773

[22] Becht E , Giraldo NA , Lacroix L , et al. Estimating the population abundance of tissue‐infiltrating immune and stromal cell populations using gene expression. Genome Biol. 2016;17: 10.1186/s13059-13016-11070-13055 PMC5134277 27908289

[23] Peng W , Chen JQ , Liu C , et al. Loss of PTEN promotes resistance to T cell‐mediated immunotherapy. Cancer Discov. 2016;6:202–216. 26645196 10.1158/2159-8290.CD-15-0283 PMC4744499

[24] Haq R , Shoag J , Andreu‐Perez P , et al. Oncogenic BRAF regulates oxidative metabolism via PGC1alpha and MITF. Cancer Cell. 2013;23:302–315. 23477830 10.1016/j.ccr.2013.02.003 PMC3635826

[25] Gopal YN , Rizos H , Chen G , et al. Inhibition of mTORC1/2 overcomes resistance to MAPK pathway inhibitors mediated by PGC1alpha and oxidative phosphorylation in melanoma. Can Res. 2014;74:7037–7047. 10.1158/0008-5472.CAN-14-1392 PMC4347853 25297634

[26] Najjar YG , Menk AV , Sander C , et al. Tumor cell oxidative metabolism as a barrier to PD‐1 blockade immunotherapy in melanoma. JCI. Insight. 2019;4: 10.1172/jci.insight.124989 PMC6483505 30721155

[27] Schadendorf D , Long GV , Stroiakovski D , et al. Three‐year pooled analysis of factors associated with clinical outcomes across dabrafenib and trametinib combination therapy phase 3 randomised trials. Eur J Cancer. 2017;82:45–55. 28648698 10.1016/j.ejca.2017.05.033

[28] Joseph RW , Elassaiss‐Schaap J , Kefford R , et al. Baseline Tumor Size Is an Independent Prognostic Factor for Overall Survival in Patients with Melanoma Treated with Pembrolizumab. Clin Cancer Res. 2018;24:4960–4967. 29685882 10.1158/1078-0432.CCR-17-2386 PMC6916264

[29] Wagner NB , Forschner A , Leiter U , Garbe C , Eigentler TK . S100B and LDH as early prognostic markers for response and overall survival in melanoma patients treated with anti‐PD‐1 or combined anti‐PD‐1 plus anti‐CTLA‐4 antibodies. Br J Cancer. 2018;119:339–346. 29950611 10.1038/s41416-018-0167-x PMC6070917

[30] Huang AC , Postow MA , Orlowski RJ , et al. T‐cell invigoration to tumour burden ratio associated with anti‐PD‐1 response. Nature. 2017;545:60–65. 28397821 10.1038/nature22079 PMC5554367

[31] Cascone T , McKenzie JA , Mbofung RM , et al. Increased tumor glycolysis characterizes immune resistance to adoptive T cell therapy. Cell Metab. 2018;27:977–987. 29628419 10.1016/j.cmet.2018.02.024 PMC5932208

[32] Micevic G , Theodosakis N , Bosenberg M . Aberrant DNA methylation in melanoma: biomarker and therapeutic opportunities. Clinical. Epigenetics. 2017;9: 10.1186/s13148-13017-10332-13148 PMC5381063 28396701

[33] Van Allen EM , Miao D , Schilling B , et al. Genomic correlates of response to CTLA‐4 blockade in metastatic melanoma. Science. 2015;350:207–211. 26359337 10.1126/science.aad0095 PMC5054517

[34] Hu E‐C , He J‐G , Liu Z‐H , et al. High levels of serum lactate dehydrogenase correlate with the severity and mortality of idiopathic pulmonary arterial hypertension. Experimental and Therapeutic Medicine. 2015;9:2109–2113. 26136943 10.3892/etm.2015.2376 PMC4473598

[35] Nielsen VK , Kemp E , Laursen T . Lactic dehydrogenase in kidney tissue and renal disease. Adaptive change of the synthesis in acute failure. Acta Medica Scandinavica. 1968;184:109–119. 5703958 10.1111/j.0954-6820.1968.tb02430.x

[36] Levi M . Cancer‐related coagulopathies. Thromb Res. 2014;133(Suppl 2):S70–75. 24862149 10.1016/S0049-3848(14)50012-6

[37] Yoo H , Jung E , Gwak HS , Shin SH , Lee SH . Surgical outcomes of hemorrhagic metastatic brain tumors. Cancer Research and Treatment. 2011;43:102–107. 21811426 10.4143/crt.2011.43.2.102 PMC3138913

