# Supplementary figures and images for: Molecular and immunological associations of elevated serum lactate dehydrogenase in metastatic melanoma patients: A fresh look at an old biomarker
Source: Cancer Med. 2020 Oct 5;9(22):8650–61. doi: 10.1002/cam4.3474 (PMC7666738; doi:10.1002/cam4.3474)

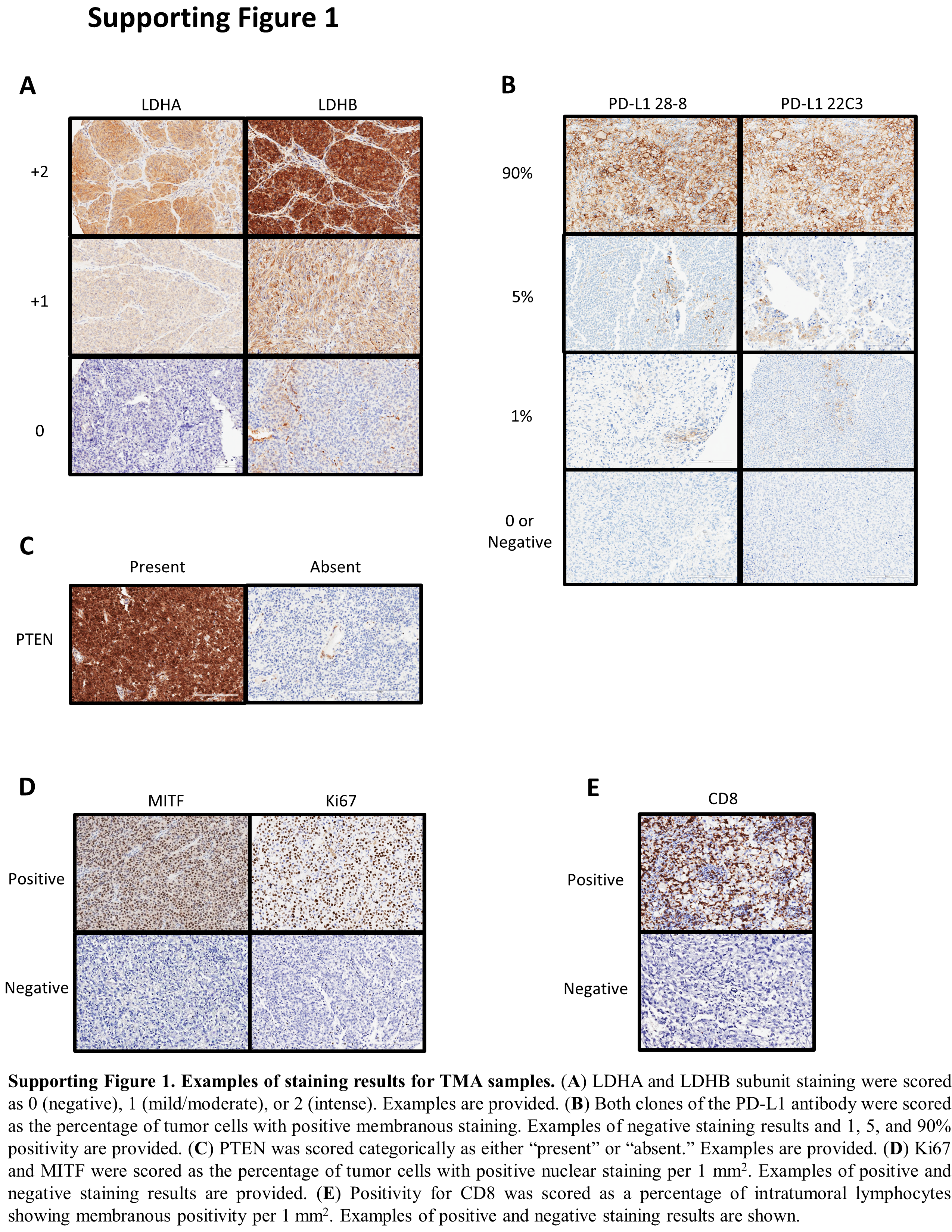

Supplement: Supplementary file 1 — Fig S1 [file CAM4-9-8650-s001.tif]

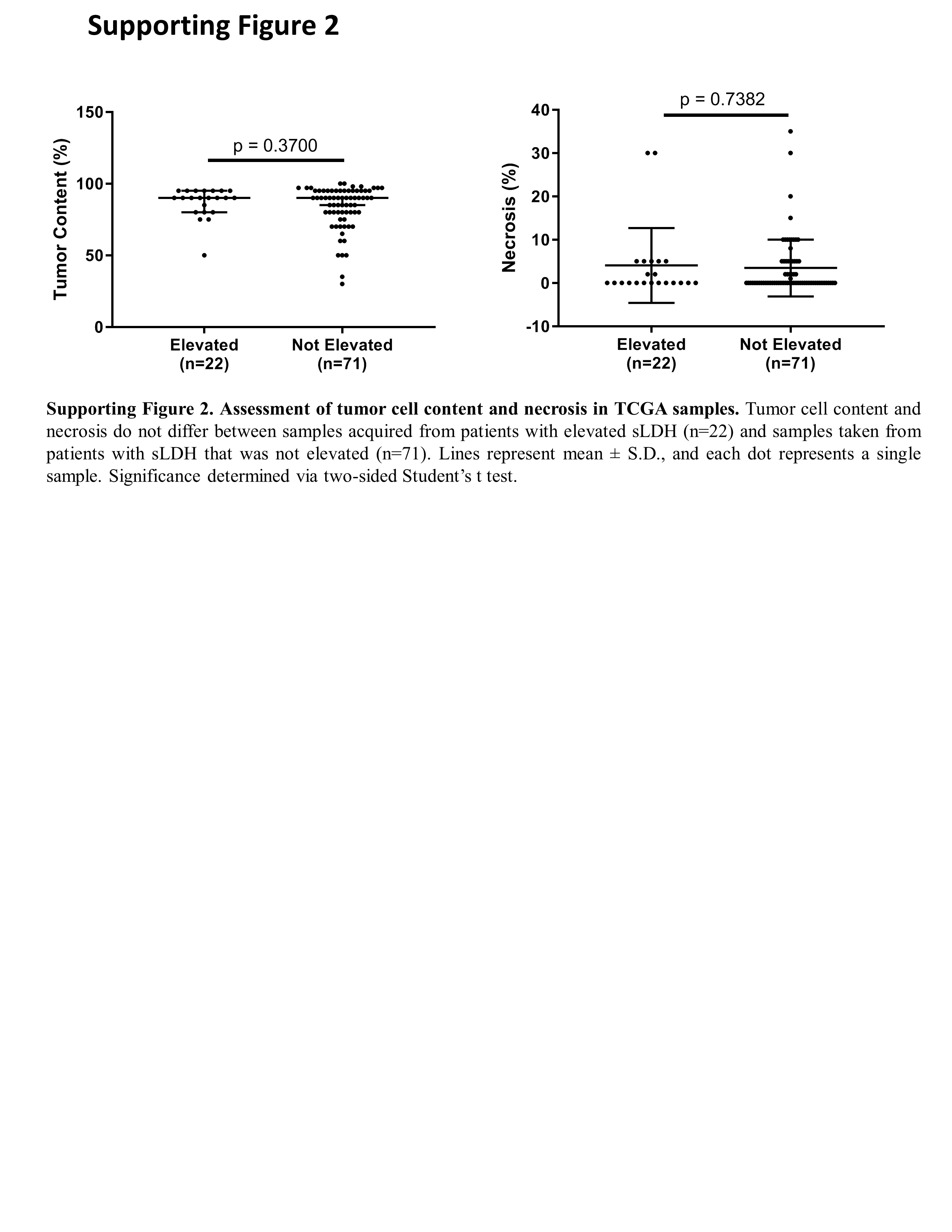

Supplement: Supplementary file 2 — Fig S2 [file CAM4-9-8650-s002.tif]

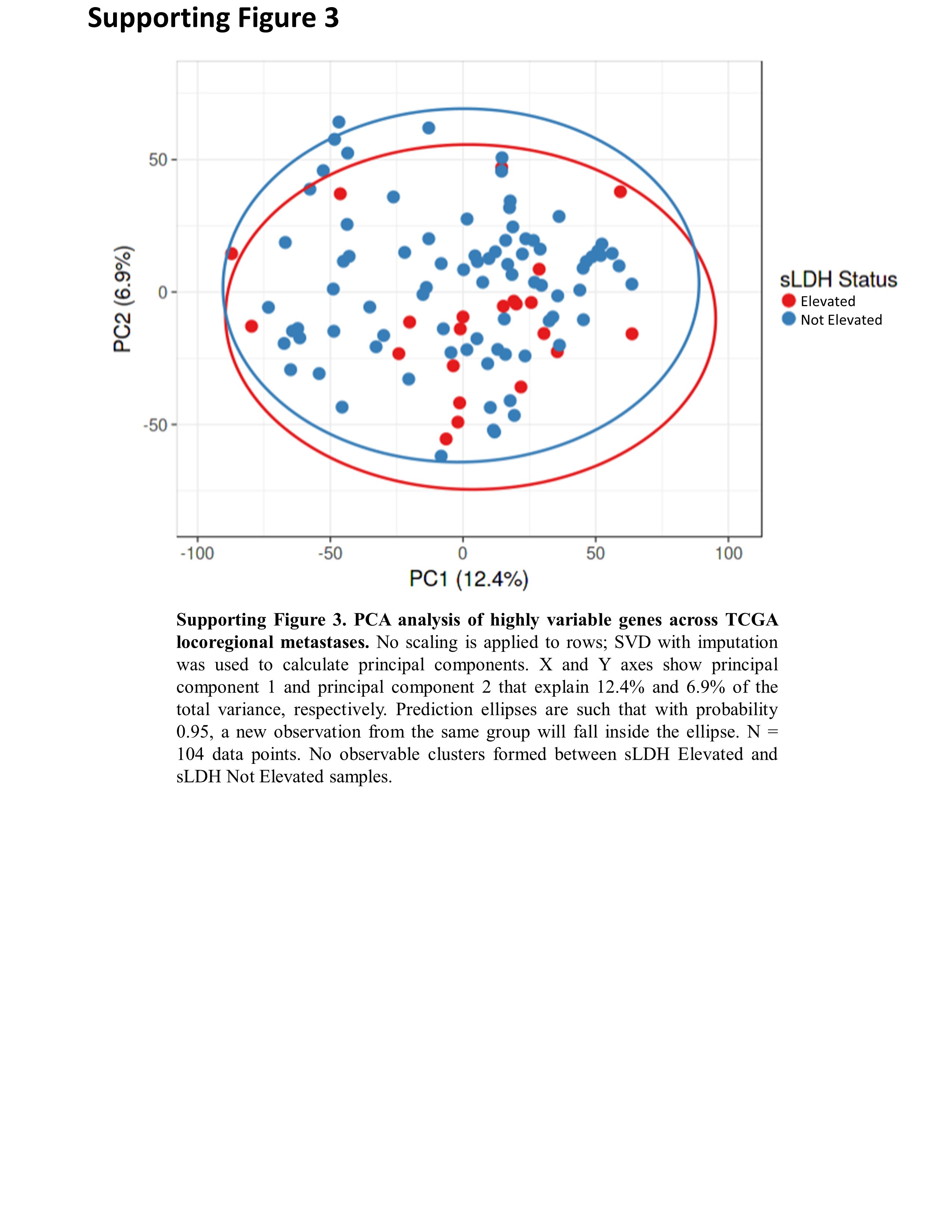

Supplement: Supplementary file 3 — Fig S3 [file CAM4-9-8650-s003.tif]

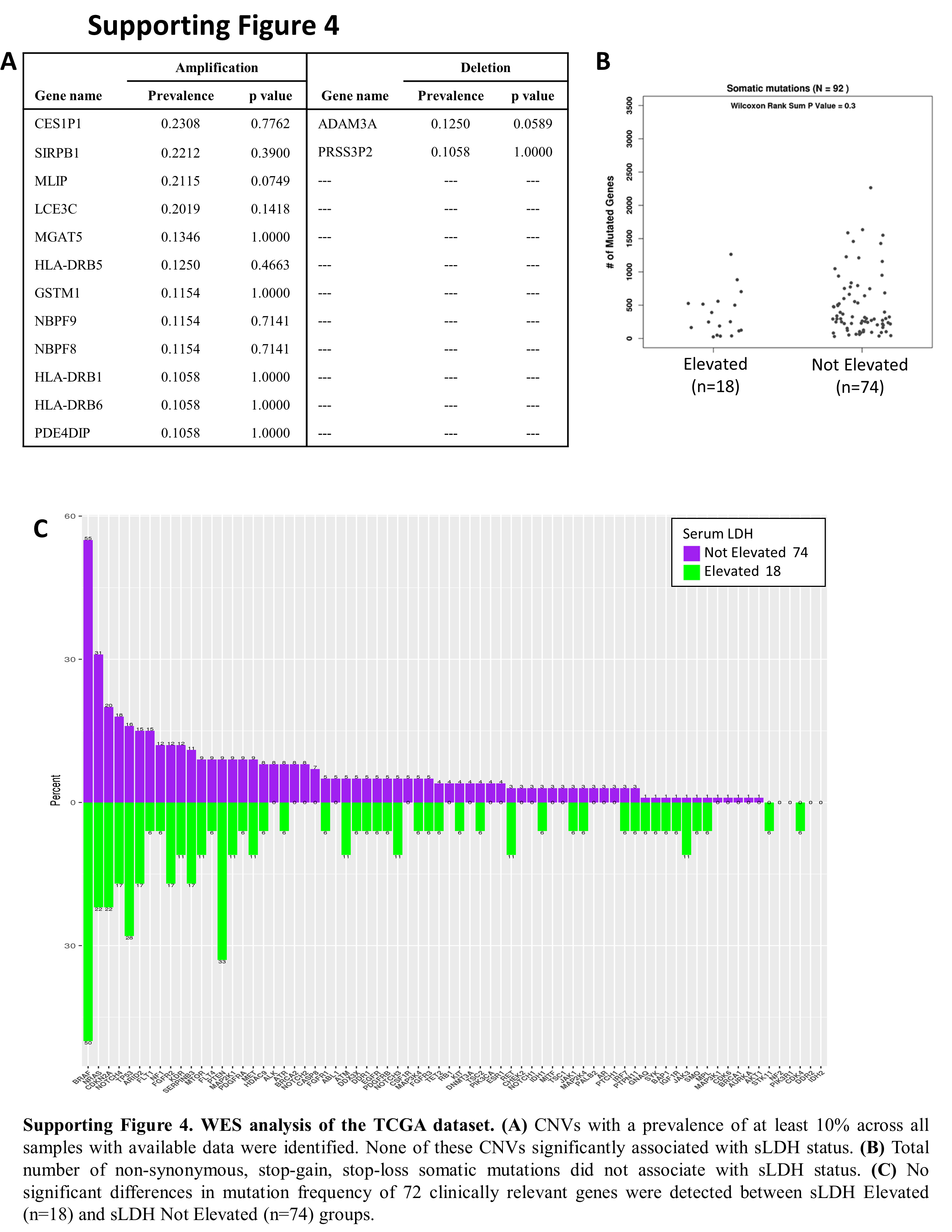

Supplement: Supplementary file 4 — Fig S4 [file CAM4-9-8650-s004.tif]

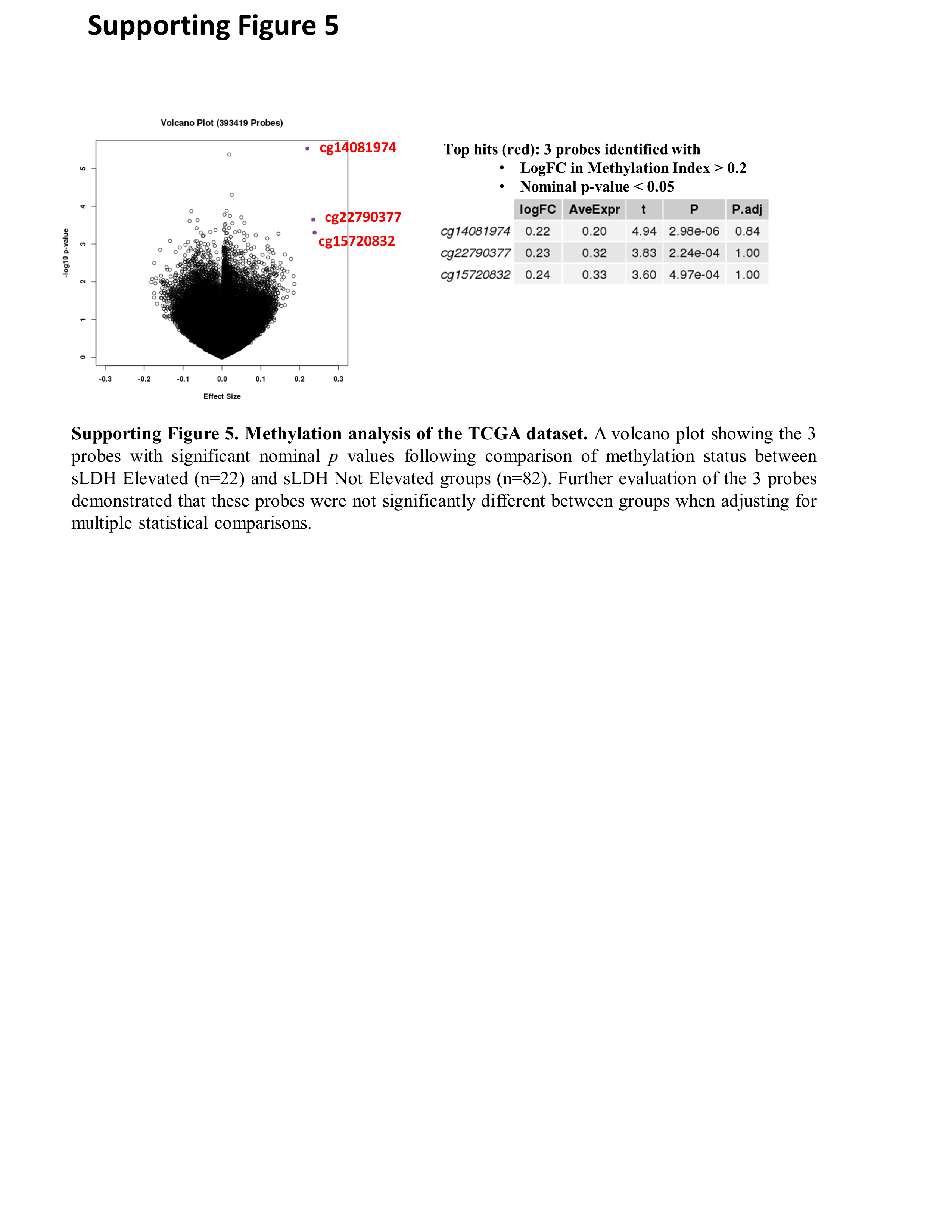

Supplement: Supplementary file 5 — Fig S5 [file CAM4-9-8650-s005.tif]

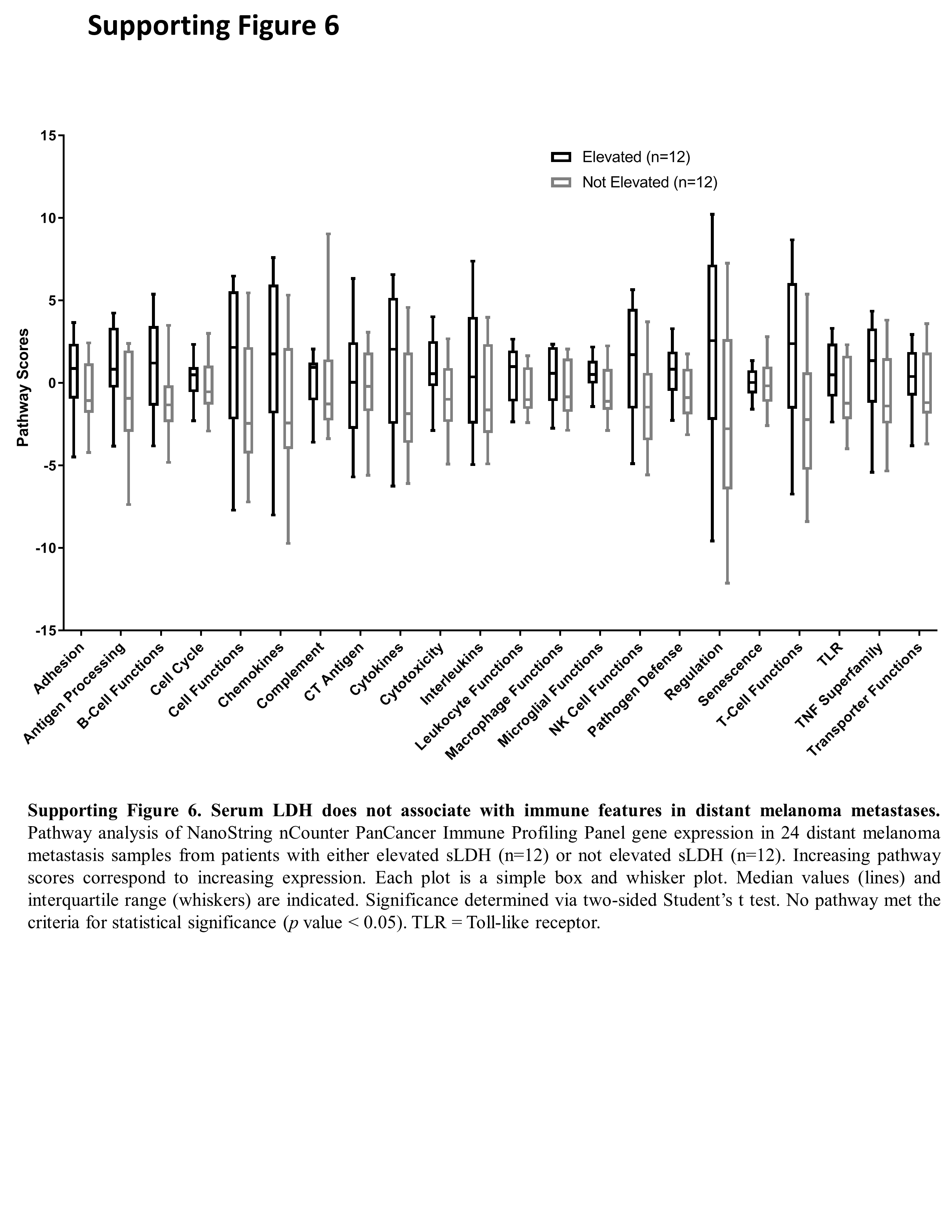

Supplement: Supplementary file 6 — Fig S6 [file CAM4-9-8650-s006.tif]

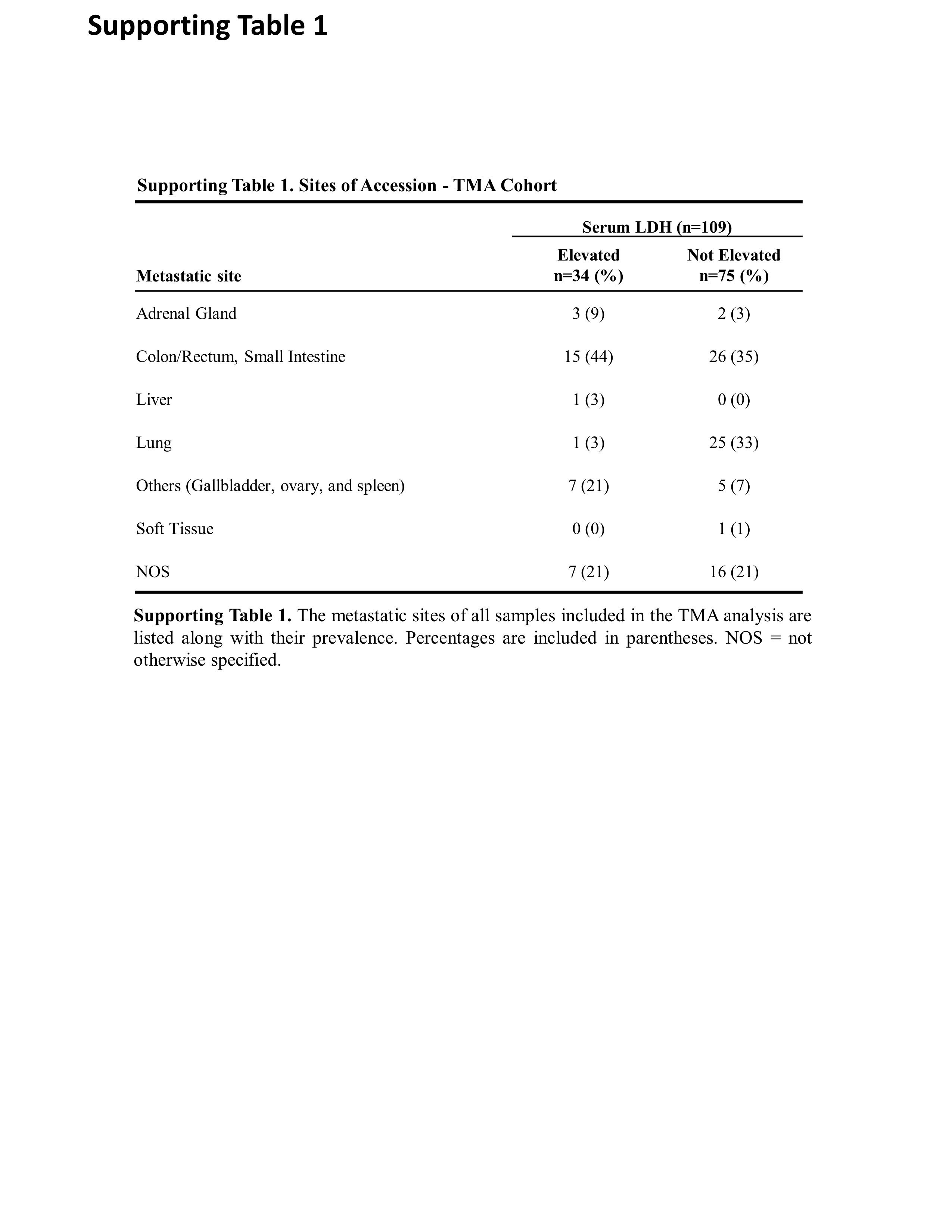

Supplement: Supplementary file 7 — Table S1 [file CAM4-9-8650-s007.tif]

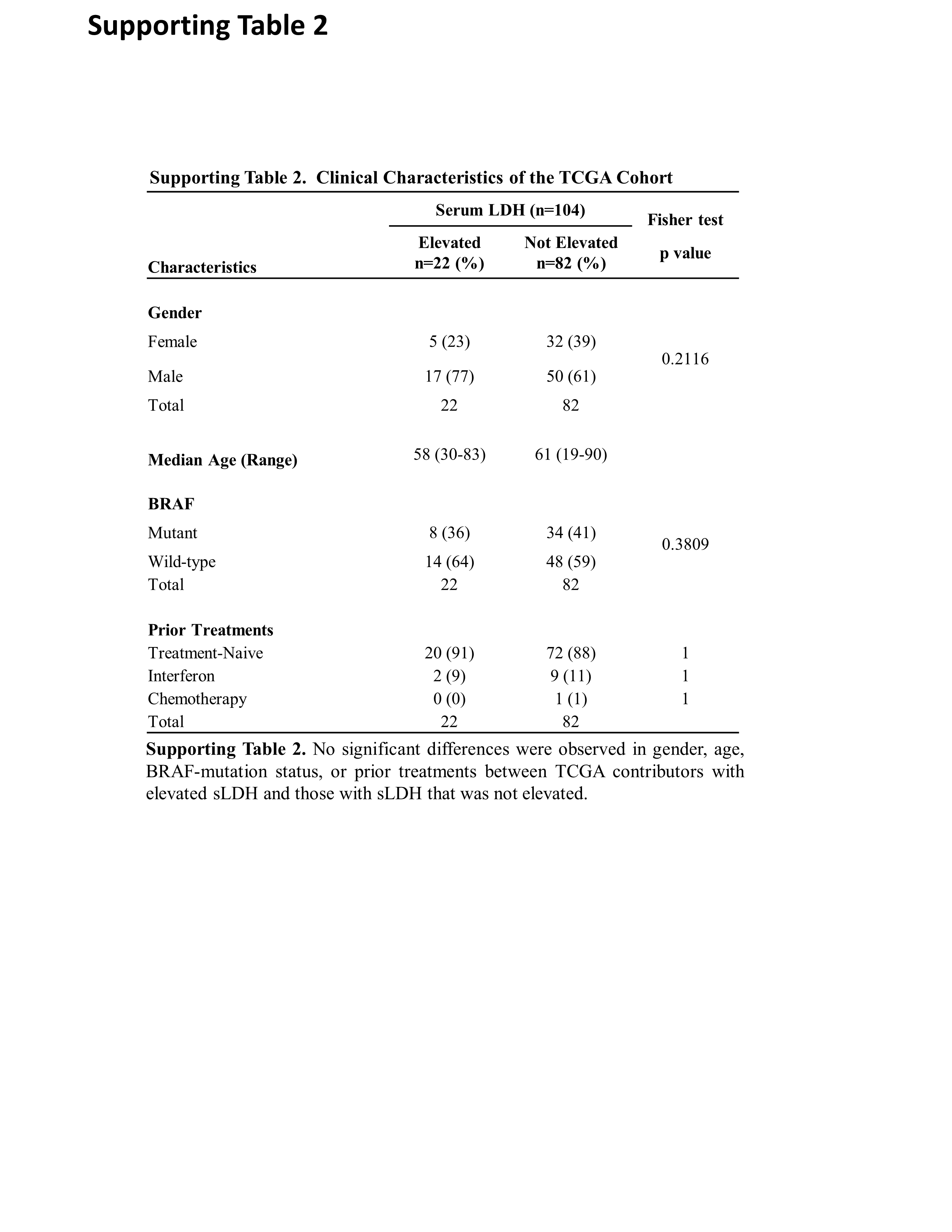

Supplement: Supplementary file 8 — Table S2 [file CAM4-9-8650-s008.tif]

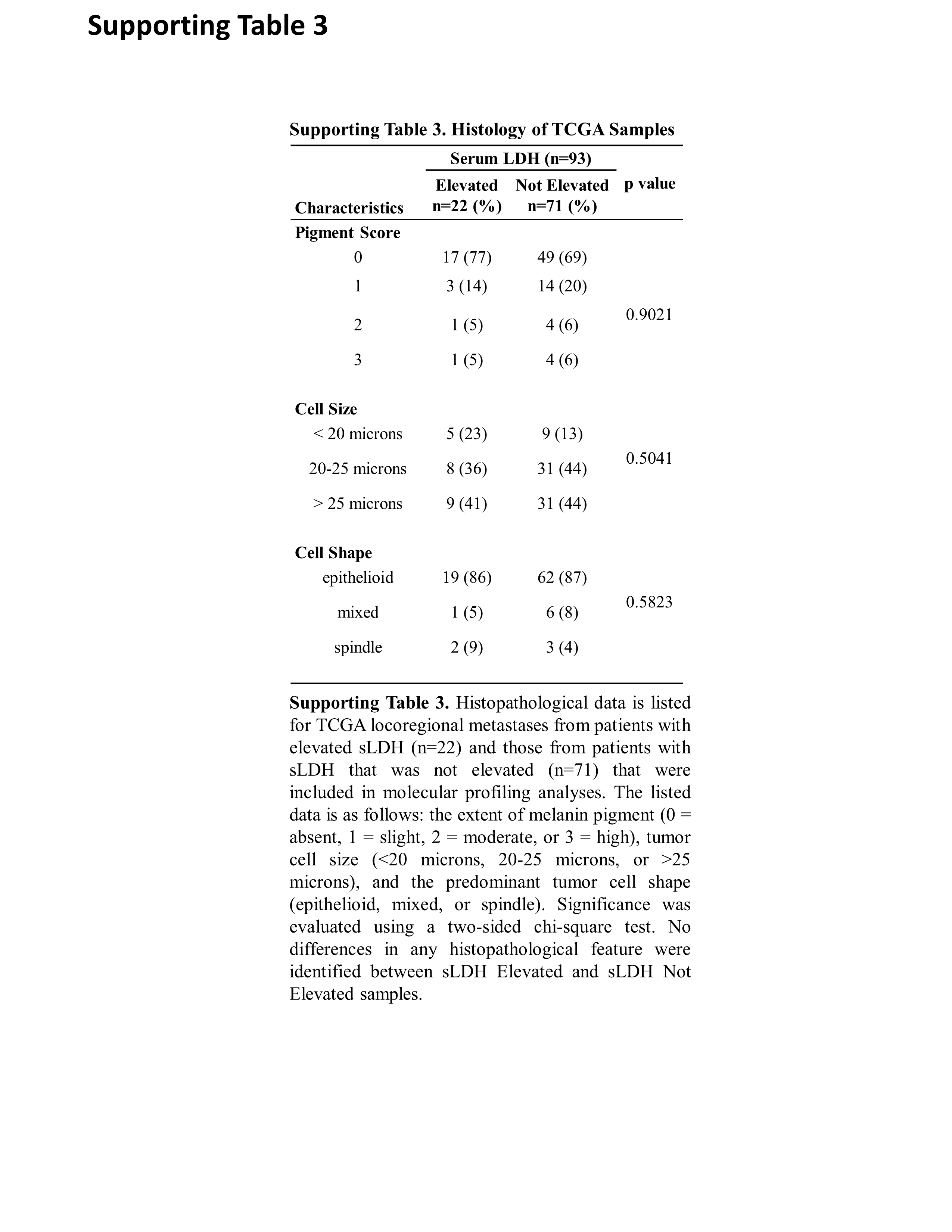

Supplement: Supplementary file 9 — Table S3 [file CAM4-9-8650-s009.tif]

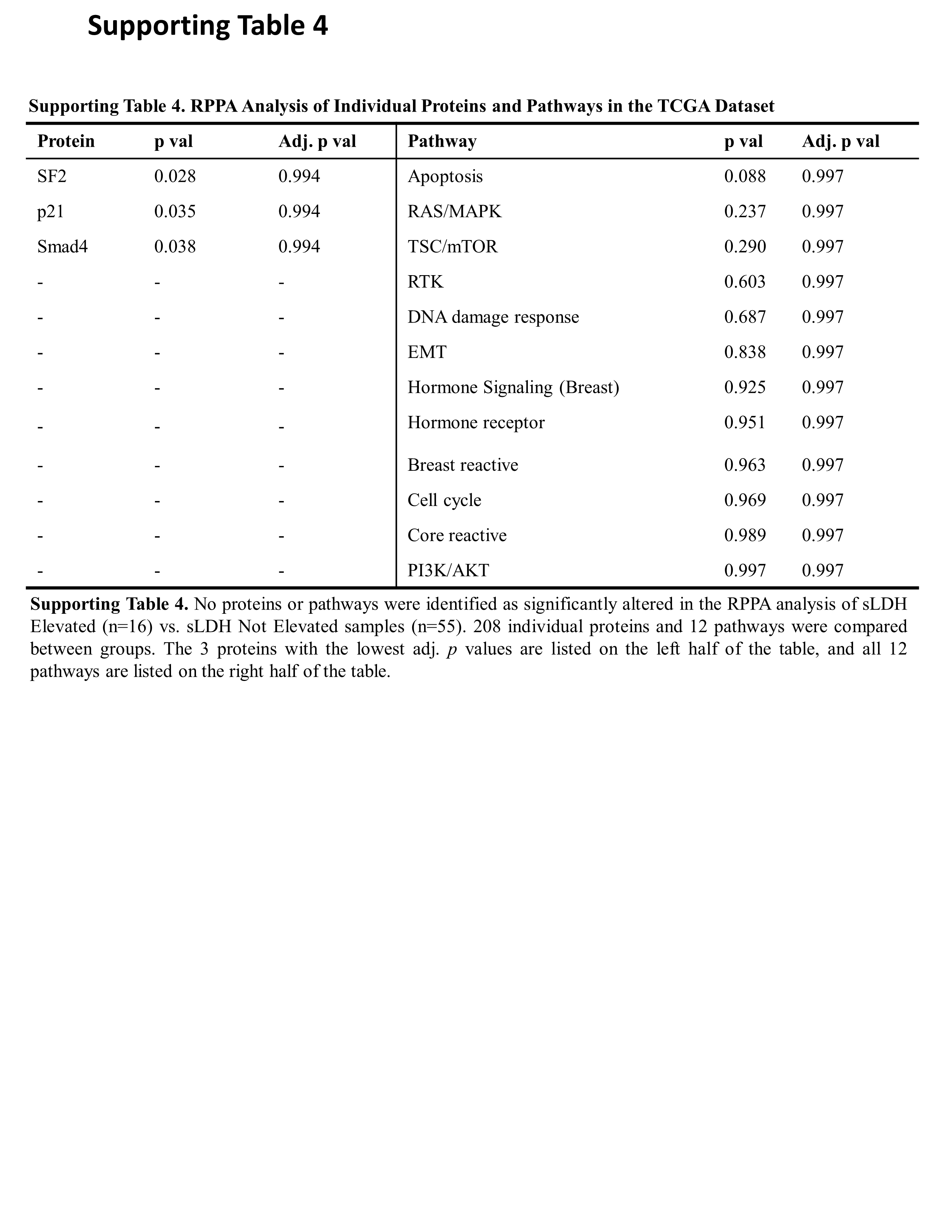

Supplement: Supplementary file 10 — Table S4 [file CAM4-9-8650-s010.tif]

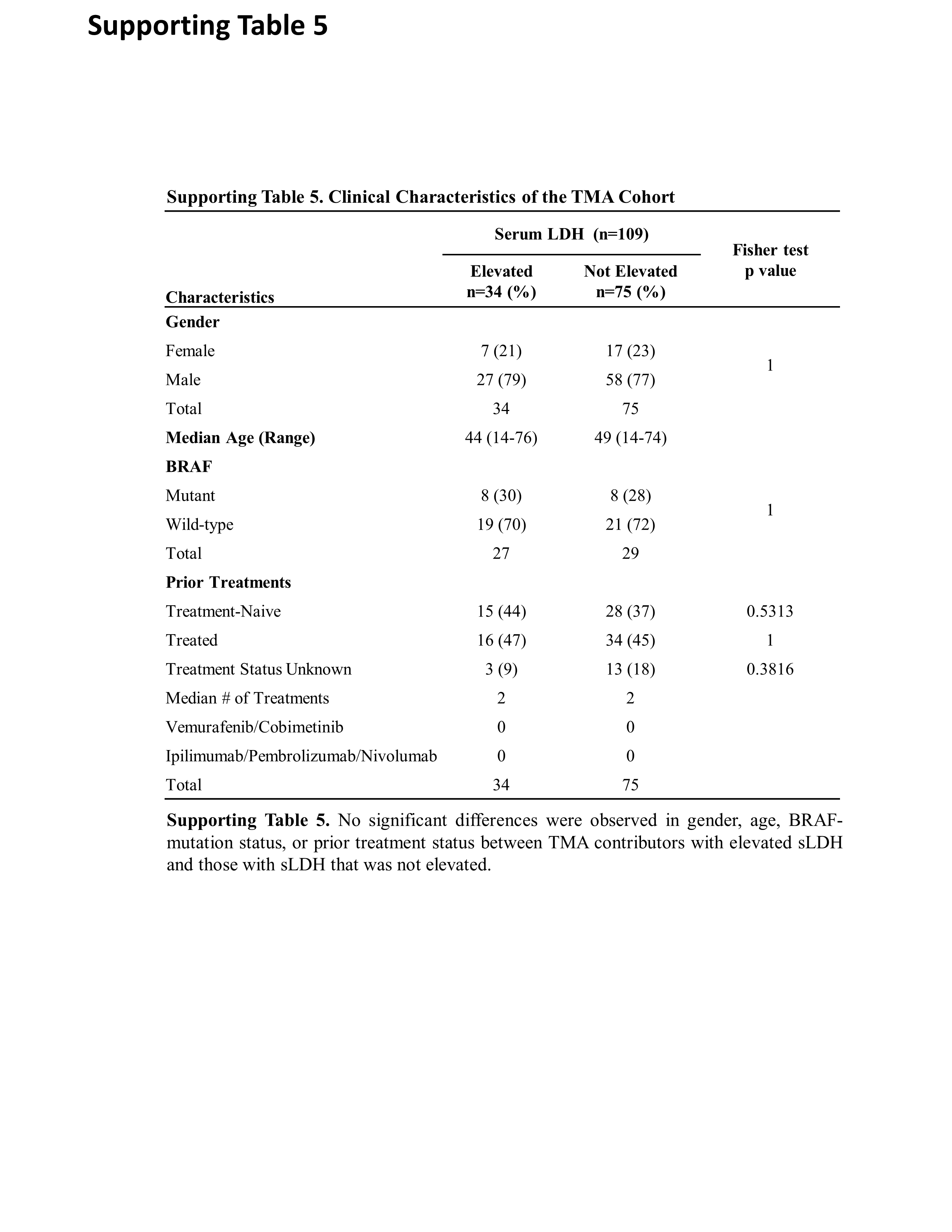

Supplement: Supplementary file 11 — Table S5 [file CAM4-9-8650-s011.tif]
