# Supplementary material for: Molecular and immunological associations of elevated serum lactate dehydrogenase in metastatic melanoma patients: A fresh look at an old biomarker
Source: Cancer Med. 2020 Oct 5;9(22):8650–61. doi: 10.1002/cam4.3474 (PMC7666738; doi:10.1002/cam4.3474)
Supplement: Supplementary file 12 — Supplementary Material [file CAM4-9-8650-s012.docx]

**SUPPLEMENTAL METHODS**

**RNA-sequencing (RNA-seq)**

Raw expected counts generated via RSEM were downloaded from GDAC Firehose for all locoregional TCGA samples with available sLDH values (Elevated, n=22; Not Elevated, n=82). Counts were normalized using the TMM method and generalized linear models were used for differential expression analysis.^1^ Comparisons of interest were performed using functions from the *edgeR* and *limma/voom* Bioconductor packages in R (v3.6.1).^1^ Lowly expressed genes were filtered from 20,531 Entrez genes to 16,434 Entrez genes using the *filterByExpr* function.^1^ Differentially expressed genes (DEGs) were defined as those genes with a │log2FC│ > 1 and a Benjamini-Hochberg corrected p value < 0.05. Pathways were assessed using the GenePattern module *GSEA* (v19.0.25)^2^ on default settings, and the voom-transformed expression matrix was used as the initial input. Immune cell infiltrates were estimated from voom-transformed counts via the ESTIMATE^3^ and MCP-Counter^4^ R packages, as previously described. Principal components analysis was performed via ClustVis (https://biit.cs.ut.ee/clustvis/). Singular value decomposition (SVD) with imputation was used to calculate principal components from median-centered, voom-transformed values of the 1,000 most variable genes.

**Point Mutations**

TCGA Level 3 somatic mutation data were downloaded from GDAC Firehose and matched to 92 locoregional metastases with available sLDH levels (Elevated, n=18; Not Elevated, n=74). The total mutation burden for each sample was calculated and compared between groups using the Mann–Whitney–Wilcoxon test. The Fisher exact test was used to assess the association between the sLDH status and mutation status of 72 therapeutically targetable genes.^5^

**Copy Number Variation (CNV)**

Level 3 TCGA CNV data were downloaded from GDAC Firehose and matched to 92 locoregional metastases with available sLDH levels (Elevated, n=18; Not Elevated, n=74). A total of 21,689 genes were classified by gene-level data based on the log2-ratio of copy numbers between samples to reference samples. A histogram showed the distribution of values between -8 to 6 (positive values indicating copy number gain and negative values indicating copy number loss). The copy number data were dichotomized as high amplification (threshold of > 5) and high deletion (threshold of < 0.5). The genes with the highest prevalence (> 10%) of copy number amplification (or deletion) were identified. Fisher’s exact test was used to test for independence between sLDH status and high amplification (or deletion) genes.

**Methylation**

Level 3 TCGA methylation data were downloaded from GDAC Firehose and matched to 92 locoregional metastases with available sLDH levels (Elevated, n=22; Not Elevated, n=82). Beta-values were compared between sLDH Elevated and sLDH Not Elevated groups. Analyses in identifying regions with methylation differences were performed through R packages *raflib* and *limma*, in which variances were shrunk by empirical Bayes and multiplicity adjustments were applied. Multiple hypothesis adjustment was performed via the Benjamini-Hochberg method.

**Reverse Phase Protein Array (RPPA)**

Level 3 TCGA RPPA data were downloaded from GDAC Firehose and matched to 71 locoregional metastases with available sLDH levels (Elevated, n=16; Not Elevated, n=55). In order to calculate pathway activity scores, RPPA data were median-centered and normalized by standard deviation across all samples for each component to obtain the relative protein level. The pathway score was calculated as the sum of the relative protein level multiplied by its weight of all components in a particular pathway.^6^ Two-sided Student’s t-test followed by multiple hypothesis testing correction via the Benjamini-Hochberg method were performed to identify differentially expressed antibodies and pathways. A total of 280 individual proteins and 12 pathways were assessed using this methodology.^6^

**NanoString**

The following specification was selected for normalization: Overall Count: Sum of Expression Per Sample, Background: None, Sample Content: Geometric Mean of Housekeeping Genes, and Other Normalization: vsn (Variance Stabilizing Normalization). Post-normalization expression values for each sample were then log2-transformed before two-sample Student’s t-tests were used to compare gene expression between groups. Multiple hypothesis testing correction was performed via the Benjamini-Hochberg method.

To perform pathway analysis, raw data were uploaded to the nCounter Advanced Analysis add-on set to default settings. Data quality control, normalization, heatmap generation, and pathway scoring generation were performed with the software set to its default settings. Pathway scores were compared between groups via two-sample Student’s t-tests.

**References**

1. Law CW, Alhamdoosh M, Su S, Smyth GK, Ritchie ME. RNA-seq analysis is easy as 1-2-3 with limma, Glimma and edgeR. *F1000Research.* 2016;5:1408.

2. Subramanian A, Tamayo P, Mootha VK, et al. Gene set enrichment analysis: a knowledge-based approach for interpreting genome-wide expression profiles. *Proc Natl Acad Sci U S A.* 2005;102(43):15545-15550.

3. Yoshihara K, Shahmoradgoli M, Martinez E, et al. Inferring tumour purity and stromal and immune cell admixture from expression data. *Nat Commun.* 2013;4:doi:10.1038/ncomms3612.

4. Becht E, Giraldo NA, Lacroix L, et al. Estimating the population abundance of tissue-infiltrating immune and stromal cell populations using gene expression. *Genome Biol.* 2016;17(1):doi:10.1186/s13059-13016-11070-13055.

5. Chen K, Meric-Bernstam F, Zhao H, et al. Clinical actionability enhanced through deep targeted sequencing of solid tumors. *Clin Chem.* 2015;61(3):544-553.

6. Akbani R, Ng PK, Werner HM, et al. A pan-cancer proteomic perspective on The Cancer Genome Atlas. *Nat Commun.* 2014;5:3887.
